# Towards better Hebrew clickbait detection: Insights from BERT and data augmentation

**DOI:** 10.1371/journal.pone.0332342

**Published:** 2025-11-06

**Authors:** Talya Natanya, Chaya Liebeskind

**Affiliations:** Department of Computer Science, Jerusalem College of Technology, Jerusalem, Israel; PLOS: Public Library of Science, UNITED KINGDOM OF GREAT BRITAIN AND NORTHERN IRELAND

## Abstract

Clickbait headlines, designed to entice readers with sensationalized or misleading content, pose significant challenges in the digital landscape. They exploit curiosity to generate traffic and revenue, often at the cost of spreading misinformation and undermining the credibility of online content. Identifying clickbait is essential for improving the quality of information consumed, fostering trust in digital media, and enabling users to make informed decisions. This study advances Hebrew clickbait detection through deep learning approaches and comprehensive data augmentation strategies, targeting the unique challenges of processing a low-resource language. Building on prior research that achieved an accuracy of 87% using traditional machine learning methods, this work explores the potential of BERT-based models and diverse augmentation techniques to further enhance performance. Our experiments incorporated a variety of augmentation methods, including weak supervision, substitution-based methods, generative techniques and language-based methods, applied to state-of-the-art Hebrew language models. The results highlight that targeted augmentation strategies, particularly those focusing on word-level replacements and contextual enhancements, consistently improved model performance. Our top-performing configuration achieved an accuracy of 92%, surpassing traditional machine learning benchmarks. These study results can be applied in real-world systems to automatically detect and reduce clickbait in Hebrew digital media, supporting news websites and social platforms in improving content quality and user trust. Furthermore, it provides a replicable framework for tackling similar challenges in other underrepresented languages, highlighting the transformative potential of combining advanced deep learning methods with tailored data augmentation strategies.

## Introduction

The digital era has brought an explosion of information, but with has come a surge of sensationalized and misleading headlines, commonly known as clickbait. These headlines exploit curiosity and emotion to attract clicks, often at the expense of credibility and content quality. While clickbait detection has seen substantial progress in high-resource languages like English, addressing this issue in low-resource languages remains a significant challenge. Hebrew, for instance, suffers from a lack of large annotated datasets and linguistic tools, which are essential for building effective machine learning and natural language processing (NLP) models. This gap in resources necessitates innovative approaches to improve clickbait detection and ensure the reliability of online information in Hebrew.

This study aims to tackle these challenges by leveraging recent advancements in deep learning, particularly the use of BERT-based models. BERT (Bidirectional Encoder Representations from Transformers) [1] represents a significant breakthrough in natural language processing, introduced by Google in 2018. It employs a novel bidirectional training architecture that allows it to understand the context of a word based on all of its surroundings, both the words that come before and after it. This contextual understanding is achieved through a pre-training process on large text corpora using two key tasks: Masked Language Modeling (MLM), where the model predicts randomly masked words in a sentence, and Next Sentence Prediction (NSP), where it learns to understand relationships between sentences. This pre-training approach enables BERT to capture deeper and richer language representations compared to traditional unidirectional models. Hebrew-specific adaptations of BERT models, such as HeBERT [2], AlephBERTGimel [3], and DictaBERT [4], along with the multilingual mBERT model [5], offer promising avenues for improving Hebrew clickbait detection. By combining these state-of-the-art models with targeted data augmentation strategies, this research seeks to push the boundaries of what can be achieved in low-resource NLP.

Data augmentation plays a central role in this study and is divided into four main strategies: 1. Weak supervision expands the dataset by automatically labeling new examples. This approach uses the best-performing machine learning model from previous research to annotate data, allowing for rapid dataset growth with minimal manual intervention. 2. Substitution-based methods introduce variability by replacing, swapping, or removing sentence elements. These include syntactic and semantic substitutions, which generate diverse and contextually relevant training examples. 3. Generative methods leverage large language models to create synthetic headlines, enriching the dataset with high-quality, augmented samples. 4. Language-based methods utilize cross-lingual and multilingual techniques alongside round-trip translation, to incorporate knowledge from other languages. This introduces new semantic patterns and linguistic variations into the Hebrew data.

By integrating these augmentation strategies, the study demonstrates that combining advanced deep learning models with tailored data augmentation significantly improves clickbait detection in Hebrew. Our experiments highlight the effectiveness of substitution-based and generative methods, with the best-performing approach achieving an impressive 92% accuracy, significantly surpassing traditional machine learning benchmarks.

This work contributes to the growing field of low-resource NLP by systematically evaluating augmentation techniques and identifying best practices for enhancing model performance. The insights gained not only advance Hebrew clickbait detection but also provide a replicable framework for addressing similar challenges in other underrepresented languages. Through this research, we aim to foster a more reliable and trustworthy digital media ecosystem in low-resource settings.

## Background

Data augmentation for text classification is a technique specifically valuable when training models with limited labeled data. It combats this challenge by creating synthetic training examples, leading to improved model generalization, reduced class imbalances, and ultimately, better performance. Expanding the dataset size also helps mitigate overfitting, enabling models to identify more robust patterns within the data. In this literature review we explore common augmentation techniques for text classification in general, augmentation techniques specifically for clickbait detection and data augmentation for low-resource languages.

### Data augmentation for text classification

Data augmentation techniques can be applied at various levels of text granularity, including character, word, sentence, and document level, as outlined by Bayer [6] in his survey on data augmentation for text classification. However, clickbait detection heavily relies on the meaning and context conveyed through sentences and documents. Consequently, we will focus primarily on the techniques presented in the survey that are applicable at the sentence and document levels.

#### Sentence level augmentation.

- Structure-based transformation: This approach modifies the structure of existing sentences to create new ones. This can involve:
  1. Subject/object inversion [7]: Changing the grammatical structure from active to passive voice (e.g. “The dog chased the cat” -> “The cat was chased by the dog”).
  2. Semantic text exchange [8]: This method involves identifying replaceable phrases in the original text and substituting them with masked tokens. An attention-based language model then fills these tokens, ensuring the replacements align contextually with the new entities. The approach selects frequent nouns as replacement candidates and is particularly effective for short texts.
- Interpolation [9]: Constructs new data points from existing ones by replacing phrases in training examples with similar phrases from other examples that have the same POS tag and class label. For example, “the [DT] girl [NN]” can be replaced with “a [DT] story [NN]” as long as both instances share the same class label.

#### Document level augmentation.

- Round-trip translation [10]: Involves translating a word, phrase, sentence, or document into another language and then back into the source language.
- Generative methods: Generative methods leverage large language models (LLMs) such as ChatGPT, Gemini, and other advanced transformers to create synthetic text data. These methods operate by generating new examples based on prompts (for example rule-based prompting [11,12]) or by rephrasing existing data while preserving the underlying context, semantics, and meaning [11,13,14]. These methods are widely applied across various types of datasets in English [12–14] and in low-resource languages, such as Indian languages [11].

#### Easy data augmentation (EDA).

In another study [15], a set of simple yet effective techniques was introduced, tailored specifically for text classification tasks known as Easy Data Augmentation (EDA):

- Synonym replacement (SR): Randomly selects n words from a sentence (excluding stop words) and replaces each with a randomly chosen synonym. (When using SR with BERT-based models, this approach closely aligns with the Semantic Text Exchange method).
- Random insertion (RI): Inserts a randomly selected synonym of a random word (that is not a stop word) into a random position in the sentence, repeated n times.
- Random swap (RS): Swaps the positions of two randomly chosen words in the sentence, repeated n times.
- Random deletion (RD): Removes each word in the sentence with a given probability p.

### Text augmentation for clickbait detection

Data augmentation techniques have found significant application in clickbait detection studies, aiming to enrich training datasets and elevate model performance. Researchers have leveraged various methods, including those previously mentioned, such as synonym replacement from EDA. Hättasch and Binnig [16] demonstrated the effectiveness of this approach, increasing the BertScore from 0.2137 to 0.2523.

Innovative approaches have also emerged, focusing on developing deep learning models for title generation based on article content. Wei and Zou [17] introduced a sophisticated system in English, a resource-rich language, combining a document auto-encoder with a headline generator. This system creates stylistically specific headlines while maintaining document-headline relationships and style differentiability using multiple discriminators. Their approach increased accuracy from 0.692 to 0.742.

Kumar and Khattar [18] proposed an algorithm that generates new headlines by identifying key sentences in news articles. It extracts nouns from both the headlines and key sentences, swapping them to create new variations. This method significantly boosted accuracy from 0.7257 to 0.9942.

### Text augmentation for low-resource languages

Since Hebrew is a low-resource language with limited data augmentation tools, we explored data augmentation techniques for such languages. Most techniques we found use dependency trees, including:

- Dependency tree cropping [19]: Removing specific dependency links to create new sentence structures, altering meaning while preserving syntax.
- Dependency tree rotation [19]: Reordering subtrees around the root node to introduce syntactic variations.
- Nonce [20]: Generating sentences by replacing single words with compatible ones from other sentences.
- Swap [21]: Identifying and swapping subtrees within the dependency tree to rearrange sentence structure while maintaining grammatical correctness.

In Hebrew, as in other low-resource languages, parsing tools are available to support the implementation of these approaches. But, in recent years, significant efforts have been made to develop BERT models tailored specifically for Hebrew, alongside advancements in multilingual models. These developments enable us, in this article, to explore a broader range of methods and approaches applicable to low-resource languages like Hebrew.

## Clickbait detection in Hebrew

### The dataset

The dataset employed in this study is derived from a previous work on clickbait detection in Hebrew [22]. It comprises approximately 1400 headlines, evenly distributed between clickbait and non-clickbait. The labeling of the headlines was based on the clickbait definition established in the referenced study, and six distinct categories derived from this definition.

The definition of clickbait used in this context is: “clickbait refers to a headline that entices readers to seek more information through a link by manipulation, exaggeration, or dishonesty about the content of the article, while a non-clickbait headline delivers straightforward key information.” The six categories are as follows:

1. A headline that piques curiosity but fails to provide substantial information.
2. A headline that presents a fact in an exaggerated and arguable manner.
3. A headline that references an unidentified subject or entity.
4. A headline that attempts to evoke emotional reactions from readers.
5. A headline that attracts readers to seek more information in a manipulative way.
6. Non-clickbait.

A variety of machine learning models were trained on this dataset, with the results summarized in Table 1. Support Vector Machines (SVM) demonstrated the highest performance, achieving an accuracy of 0.87. In parallel, the deep learning model HeBERT, was fine-tuned, yielding a similar accuracy of 0.87.

**Table 1:** Previous research results.

Model	Accuracy	F1 Score
HeBERT	0.87	0.87
SVM	0.87	0.87
Logistic Regression	0.85	0.85
Random Forest	0.82	0.82
Bagging	0.79	0.79
Naïve Bayes	0.76	0.70

## Methods

**Fig 1:**
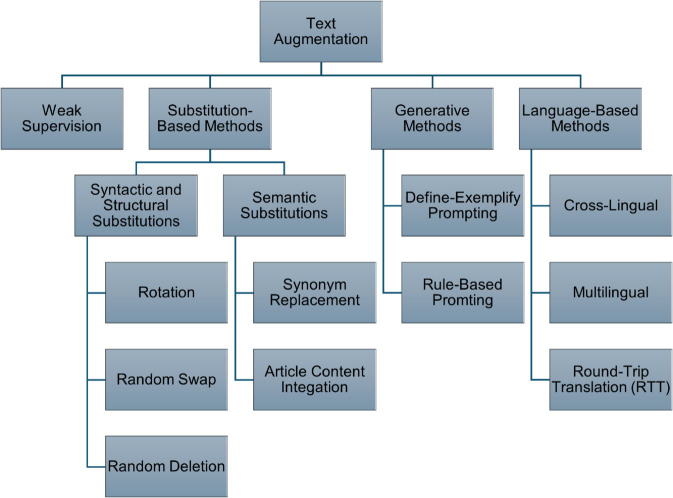
Text augmentation techniques schema.

Given the typical superiority of deep learning models on larger datasets, this study explores various text augmentation techniques, as illustrated in Fig 1. The process begins with weak supervision, followed by substitution-based methods (semantic, syntactic and structural), generative approaches, and concludes with language utilization methods. The schema in Fig 1 supports multiple possible strategies, such as applying augmentation techniques in parallel, combining them through contrastive learning, or structuring them in layered pipelines. In this study, we applied each augmentation method individually in order to isolate its specific contribution. Future work may explore contrastive learning or combined augmentation strategies to further improve model robustness and performance.

### Weak supervision

In our initial data augmentation experiment, we employed a weak supervision approach, as suggested in previous research on Hebrew clickbait detection [22]. Weak supervision, while closely related to semi-supervised learning, differs in that it relies on noisy or heuristic-based labeling functions. These functions are typically derived from existing models or predefined rules, rather than from a small set of high-quality labeled data that guides the learning of unlabeled examples, as in semi-supervised learning.

In this study, we collected additional headlines from the same news websites as the original dataset: Mako (https://www.mako.co.il/), N12 (https://www.n12.co.il/), Ma’ariv (https://www.maariv.co.il/) and Walla (https://www.walla.co.il/) and used the Support Vector Machine (SVM), the best-performing machine learning model from preliminary experiments, to automatically label them. This approach allowed us to significantly expand the dataset size, increasing it by up to fivefold, while minimizing the need for manual annotation and improving the model’s generalization capabilities.

### Substitution-based methods

As mentioned before, sentence- and document-level augmentations are most relevant for clickbait detection, as they preserve contextual meaning. Substitution-based methods were chosen to introduce controlled variability into the text while maintaining its semantic core. This approach is particularly suitable for Hebrew due to its rich morphology and flexible syntax.

#### Syntactic and structural substitution.

**Fig 2:**
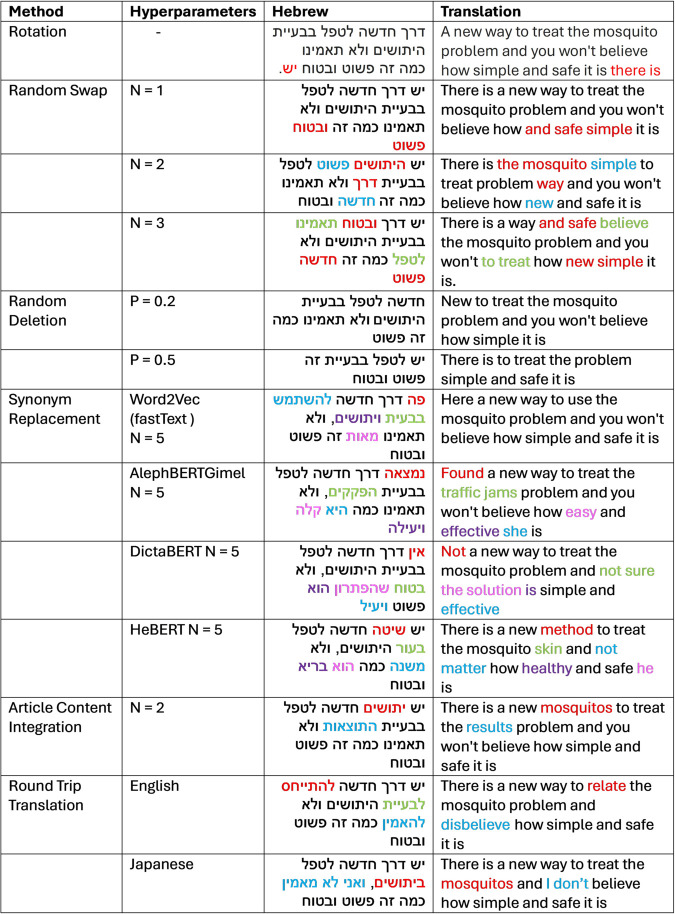
Examples of sentence augmentation across different methods. Figure 2 showcases the application of the various methods on a sample sentence from the original dataset: “y/s drK .hd/sh l.tpl bb‘yyt hytw/sym wl’ t’mynw kmh zh p/sw.t wb.tw.h” (There is a new way to treat the mosquito problem and you won’t believe how simple and safe it is).

- **Rotation**: We utilized a Hebrew parser [23] to perform syntactic substitutions through dependency tree rotation, rearranging sentence structures by rotating syntactic elements within the dependency tree. For each sentence, we generated four substitutions (rotates), where each substitution produced a new variation of the original sentence. As a result, the dataset was expanded up to fivefold. However, it’s important to note that the resulting augmented sentences are not guaranteed to be syntactically correct (like the placement of the words “there is” in the example provided in Fig 2).
- **Random swap (RS)**: We then implemented the EDA Random Swap (RS) method, selecting N random words (N = 1, 2, 3) from each sentence and replacing them with other words in the sentence that shared the same part-of-speech tag. This process of selecting and replacing N words was repeated four times, generating four additional variations for each sentence.
- **Random deletion (RD)**: The EDA random deletion (RD) method was also implemented, where words were omitted from each sentence with a probability P ranging from 0.2 to 0.5.

#### Semantic substitutions.

- **Synonym replacement (SR)**: For the EDA synonym replacement (SR) method, we replaced N words in each sentence with their synonyms (N = 3, 4, 5). For each word, four different synonyms were provided, ranked by semantic similarity. In the first increment, the top synonym for each word was used; in the second, the second-ranked synonym, and so on, generating four new versions of the original headline. (The choice of N was guided by the headlines statistics: the shortest headline contains three words, the average headline length is eight words, and the average number of stop words per headline is two.)
  We explored three approaches for obtaining synonyms:
  1. **WordNet**: A lexical database grouping words into sets of cognitive synonyms, previously used for data augmentation in clickbait detection tasks [16]. However, the Hebrew WordNet [24] has demonstrated limited coverage [25], making it less effective for our purposes.
  2. **Word2Vec (W2V)**: Word2Vec [26] generates vector representations of words, enabling synonym identification based on vector similarity. For this study, we employed fastText [27], an extension of the Word2Vec model, which offers additional capabilities. Word2Vec treats each word in the corpus as an atomic entity, generating a single vector representation for each word. In contrast, fastText operates at a more granular level by incorporating character n-grams. This means that words are represented as the sum of their character n-gram vectors, allowing the model to capture subword information.
    This granular approach makes fastText particularly well-suited for low-resource languages like Hebrew, where morphological variations and complex word structures are common, allowing for better handling of rare words and morphological variations. FastText implementation is available for many languages, including Hebrew [28] (https://fasttext.cc/docs/en/crawl-vectors.html).
    Notably, as illustrated in the example in Fig 2, fastText occasionally replaces a word with one of its inflected forms, reflecting its focus on subword-level representations.
  3. **BERT-based models**: These contextualized language models generate synonyms based on the entire sentence context. We employed a masked token prediction approach, where the N words in the sentence were replaced with the <mask> token, and the model predicted the most contextually suitable replacements. In this approach, we used recent Hebrew BERT-based models including AlephBERTGimel, DictaBERT, and HeBERT, both for classification and for finding synonyms. This experimental setup allows us to compare the effectiveness of BERT-based models in both roles and to determine whether using the same model for both tasks or a different model for each task yields better results.
- **Article-content integration**: We tested a method from previous clickbait augmentation research [18] that generates new headlines by identifying key sentences in news articles using cosine similarity between sentence embeddings and headline embeddings. From the most similar sentence and the original headline, N nouns (N = 1, 2, 3, 4) were extracted and randomly swapped to create new headlines. We hypothesized that incorporating the article text using this approach could lead to performance improvements.

### Generative methods

**Fig 3:**
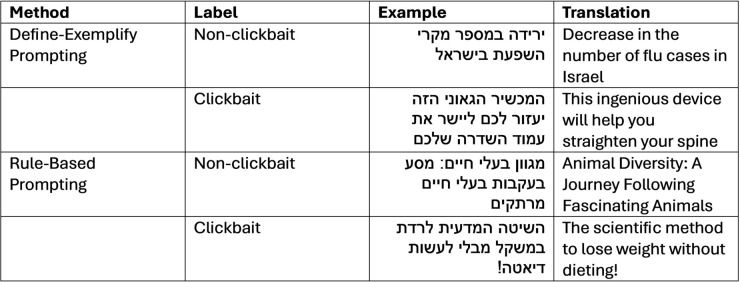
LLM’s generated examples.

**Fig 4:**
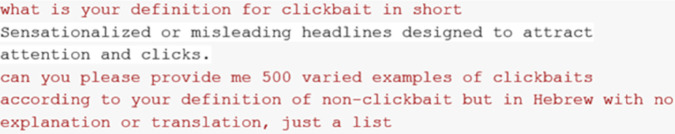
LLM’s clickbait definition.

**Fig 5:**
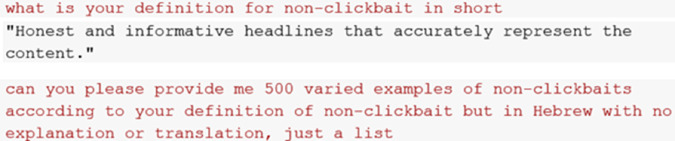
LLM’s non-clickbait definition.

**Fig 6:**
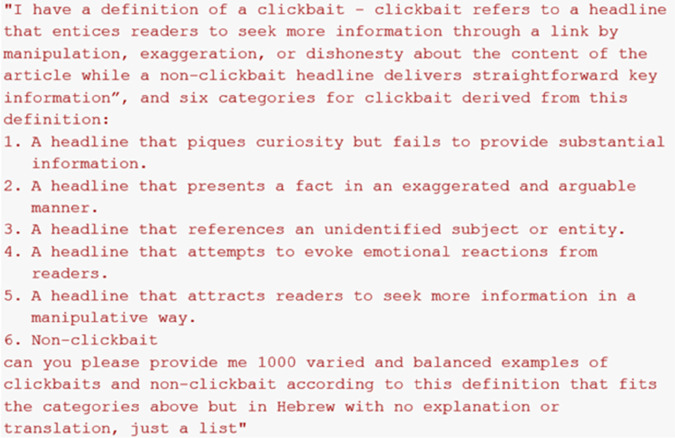
Rule-based prompting.

The recent widespread use of large language models (LLMs) for a variety of tasks, including text augmentation, has shown promising results in both high-resource languages like English [12–14] and in low-resource languages, such as Indian languages [11]. Inspired by this trend, we leveraged the Gemini LLM model (gemini-1.0-pro) to augment our dataset, experimenting with two approaches (see examples in Fig 3):

- **Define-exemplify prompting**: This approach involves asking the model to first define a concept and then generate examples based on its own definition. We asked the model to define clickbait (Fig 4) and non-clickbait (Fig 5), and then generate headlines based on its definitions.
- **Rule-based prompting**: In this approach, the model is guided by explicit rules or categories provided by the user, ensuring that generated outputs align with specific semantic or structural constraints. We supplied the model with the clickbait definition and six categories from previous research on Hebrew clickbait detection [22] and then instructed it to generate headlines based on these criteria (Fig 6).

### Language-based methods

In this set of experiments, language-based methods were chosen to leverage multilingual data resources and introduce additional semantic diversity. Given the limited availability of Hebrew data, incorporating patterns from high-resource languages or those that are structurally or typologically similar aimed to enhance the model’s ability to detect generalized clickbait cues.

#### Cross-lingual and multilingual learning.

To evaluate the effectiveness of cross-lingual and multilingual learning, we used eight diverse clickbait datasets from public sources like Kaggle and GitHub, in languages including English [29], German, Romanian [30], Bengali [31], Indonesian [32], Arabic [33], Turkish and Chinese [34] (see S1_Appendix). These datasets were chosen for their structural similarity to the Hebrew clickbait dataset. Each dataset contains between 10,000 and 70,000 labeled samples (Table 2), which were split into an 80-20 train-test, ensuring balanced label distributions.

**Table 2:** Languages dataset sizes.

Language	Samples
German	69,397
English	32,000
Turkish	20,038
Chinese	16,656
Arabic	15,473
Bengali	15,406
Indonesian	15,000
Romanian	10,866

We conducted these experiments using the Multilingual BERT (mBERT) model [5], which was pre-trained on 104 languages, covering all our explored languages.

- **Cross-lingual learning** To assess a cross-lingual model’s ability to detect clickbait in Hebrew, we trained the mBERT model individually on each foreign language dataset and evaluated it on the Hebrew test set. This approach aimed to determine whether cross-lingual knowledge could aid Hebrew clickbait detection.
- **Multilingual learning** In this phase, we evaluated whether training the mBERT on multiple languages simultaneously could improve the model’s clickbait detection in Hebrew by helping it learn language-agnostic clickbait characteristics. We tested various multilingual training strategies to see if adding multiple languages to training would enhance performance on the Hebrew test set.

Training strategies:

1. **Language-pair training**: We trained the model on each foreign language combined with the Hebrew training set and then tested it on the Hebrew test set.
2. **All languages training (9 languages total)**: In this strategy, the model was trained on all eight languages, including Hebrew, to provide a comprehensive multilingual foundation and then was tested on the Hebrew test set.
3. **Ablation test**: To assess the individual contribution of each language to the model’s performance we employed an ablation test, training the model on all languages except one. With this test we aimed to determine how the exclusion of a specific language impacts the model’s ability to detect Hebrew clickbait.

#### Round-trip translation (RTT).

Within this category of methods, we also experimented with the round-trip translation (RTT) approach. This involved translating the data into four different languages: Spanish, Japanese, French, and English, and then back to Hebrew using the Google Translate API (https://cloud.google.com/translate/docs/reference/rest). Each translation cycle introduced subtle variations between the original and translated headlines, and with each round, an additional language was incorporated. (Augmented examples are provided in Fig 2)

## Results and discussion

Building on the previous study on clickbait detection in Hebrew [22], which achieved an accuracy of 0.87 on the original dataset using the HeBERT model, we initiated our work by testing the various text augmentation methods on the same dataset with the HeBERT model. Our goal is to determine the extent of improvement these methods could provide and assess whether the results are statistically significant.

### HeBERT results

HeBERT [2] is a foundational Hebrew pretrained language model based on Google’s BERT architecture, utilizing the BERT-base configuration. HeBERT was trained on a comprehensive dataset, including the Hebrew version of OSCAR (10 GB of text, 20 million sentences), the Hebrew Wikipedia dump (650 MB of text, 3.8 million sentences), and a domain-specific dataset of emotional user-generated content (150 MB of data, 350K sentences). HeBERT serves as a reliable baseline for a range of natural language processing tasks in Hebrew, such as text classification, sentiment analysis, and question answering.

The HeBERT model showcased consistent performance across all text augmentation experiments, with notable variations based on the methods applied, as summarized in Table 3. On the original dataset, the model achieved an accuracy of 0.87 using headlines alone and 0.88 when incorporating the full content (headlines + text). The highest accuracy, 0.91, was achieved with the rule-based prompting generative method, where the dataset was expanded fivefold. This improvement was statistically significant compared to the original 0.87 accuracy, as confirmed by McNemar’s test [35] at the 0.05 signific ance level. For additional validation, we also applied the paired t-test [36] and the Wilcoxon signed-rank test [37], both of which confirmed statistical significance at the 0.05 level, marking it as the most effective approach.

**Table 3:** HeBERT results.

Method	Variant	Accuracy	F1 Score	Hyperparameters
Original data	Headlines only	0.87	0.87	-
	With full content	0.88	0.88	-
Weak supervision	-	0.86	0.86	No improvement
Rotation	-	0.89	0.89	dataX2
Random swap (RS)	-	0.90	0.89	N=1 dataX3 or N=2 dataX2
Random deletion (RD)	-	0.89	0.89	P=0.2 dataX2
Synonym replacement (SR)	Word2Vec	0.90	0.89	N=4 dataX3
	DictaBERT	0.89	0.89	dataX2 for each N
	HeBERT	0.89	0.89	N=5 dataX2 or N=3/4 dataX4
	AlephBERTGimel	0.89	0.89	N=3/4 dataX2
Generative methods	Define-exemplify prompting	0.89	0.89	dataX3
	Rule-based prompting	0.91	0.91	dataX5
Round-trip translation	-	0.89	0.89	Spanish
Article-content integration	-	0.90	0.90	N=2 dataX3

The Hyperparameters column details the specific parameters used for each data augmentation technique. dataX refers to the number of times the data was augmented.

Among the substitution-based methods, synonym replacement (SR) using Word2Vec accomplished the best performance, reaching an accuracy of 0.90 when the dataset was tripled and 4 words were replaced. The use of BERT-based models for SR (including HeBERT, AlephBERTGimel, and DictaBERT) produced consistent results, all yielding an accuracy of 0.89 with different hyperparameter configurations.

Other methods, such as rotation, random swap (RS) (with N=1 or 2), random deletion (RD) (with P=0.2), and round-trip translation (RTT), achieved accuracy scores ranging between 0.89 and 0.90, depending on the dataset size and parameters used. These methods contributed to the overall diversity and effectiveness of the augmentation approaches.

However, weak supervision was the least effective, with an accuracy of 0.86, highlighting its limited ability to enhance performance, despite increasing the dataset size.

With the recent advancements in Hebrew language models, including the release of AlephBERTGimel [3] and DictaBERT [4], we sought to assess their impact on the performance of our clickbait detection task. To achieve this, we applied the same set of text augmentation experiments to these models, comparing their results to those obtained with HeBERT. This evaluation allowed us to examine whether newer models, with expanded vocabularies and improved architectures, could offer significant performance gains and whether specific augmentation techniques were more effective on certain models.

### AlephBERTGimel results

AlephBERTGimel [3] is a more recent Hebrew language model that builds upon the success of HeBERT. It was trained on a larger dataset, including the Hebrew version of OSCAR, the Hebrew Wikipedia dump, and Hebrew Tweets collected from the Twitter sample stream (7 GB of text, 70 million sentences). Designed to further enhance performance on a variety of Hebrew language tasks, it often exhibits improved results, especially in tasks requiring deeper language understanding [3].

As shown in Table 4, AlephBERTGimel achieved an initial accuracy of 0.90 on the original dataset using headlines alone, but this dropped to 0.86 when the full content was included. The best result was obtained using the synonym replacement (SR) method, with AlephBERTGimel employed for both synonym generation and classification. This approach, which involved replacing 4 words (N = 4) and tripling the dataset, achieved an accuracy of 0.92. Random swap (RS), with 2 words swapped per sentence and the dataset doubled (N = 2, dataX2), and round-trip translation (RTT), which added translations to Spanish, showed slight improvements, both reaching an accuracy of 0.91.

**Table 4:** AlephBERTGimel results.

Method	Variant	Accuracy	F1 Score	Hyperparameters
Original data	Headlines only	0.90	0.90	-
	With full content	0.86	0.85	-
Weak supervision	-	0.86	0.85	No improvement
Rotation	-	0.90	0.90	No improvement
Random swap (RS)	-	0.91	0.90	N=2 dataX2
Random deletion (RD)	-	0.90	0.90	No improvement
Synonym replacement (SR)	Word2Vec	0.91	0.91	N=4 dataX5 or N=5 dataX2
	DictaBERT	0.91	0.90	N=5 or 3 dataX4 or N=4 dataX3
	HeBERT	0.90	0.90	No improvement
	AlephBERTGimel	0.92	0.92	N=4 dataX3
Generative methods	Define-exemplify prompting	0.89	0.89	No improvement
	Rule-based prompting	0.90	0.90	No improvement
Round-trip translation	-	0.91	0.90	Spanish
Article-content integration	-	0.90	0.90	No improvement

Weak supervision delivered the lowest performance, with an accuracy of 0.86. The rest of the methods did not demonstrate any improvement and, in some cases, even led to a decrease in performance compared to the baseline. These included rotation, random deletion (RD), generative methods (both self-prompting and rule-based prompting) and article-content integration. Overall, while AlephBERTGimel excelled with targeted augmentation techniques like SR, most methods failed to significantly improve its performance.

### DictaBERT results

DictaBERT [4] is a new state-of-the art, pre-trained BERT model for modern Hebrew, specifically designed for text summarization and generation tasks. It was trained on the HeDC4 corpus (2.5 GB of text, 910K sentences) and data from various other sources, including news sites, blogs, TV and movie subtitles, novels, and more. With a parameter size similar to previous Hebrew models, DictaBERT excels at generating concise and coherent summaries, making it a valuable tool for content creation and analysis.

As presented in Table 5, DictaBERT achieved an initial accuracy of 0.91 on the original dataset using headlines alone, marking the highest baseline performance among all models. However, accuracy dropped to 0.84 when the full content was included. The highest but not statistically significant improvement for DictaBERT was observed with several augmentation methods, all reaching an accuracy of 0.92. These included rotation with a doubled dataset (dataX2), random swap (RS) with two words swapped per sentence and the dataset tripled (N = 2, dataX3), and synonym replacement (SR) under various hyperparameter configurations.

**Table 5:** DictaBERT results.

Method	Variant	Accuracy	F1 Score	Hyperparameters
riginal data	Headlines only	0.91	0.91	-
	With full content	0.84	0.83	-
Weak supervision	-	0.87	0.86	No improvement
Rotation	-	0.92	0.92	dataX2
Random swap (RS)	-	0.92	0.92	N=2 dataX3
Random deletion (RD)	-	0.91	0.90	No improvement
Synonym replacement (SR)	Word2Vec	0.92	0.92	N=3 dataX2 or N=4 dataX3
	DictaBERT	0.92	0.92	N=5 dataX2 or N=3 dataX4 or N=4 dataX5
	HeBERT	0.92	0.92	N=3 dataX2
	AlephBERTGimel	0.91	0.91	No improvement
Generative Methods	Define-exemplify prompting	0.89	0.88	No improvement
	Rule-based prompting	0.90	0.89	No improvement
Round-trip translation	-	0.91	0.91	No improvement
Article-content integration	-	0.91	0.91	No improvement

Other methods did not improve the baseline accuracy, including weak supervision (yielding the lowest accuracy at 0.87), random deletion (RD), round-trip translation (RTT), generative methods (both self-prompting and rule-based prompting), and article-content integration. Among these, weak supervision demonstrated the least effectiveness, offering no meaningful contribution to performance improvement. In summary, while DictaBERT exhibited strong performance on the original dataset, its enhancements with augmentation methods failed to provide statistically significant benefits.

**Fig 7:**
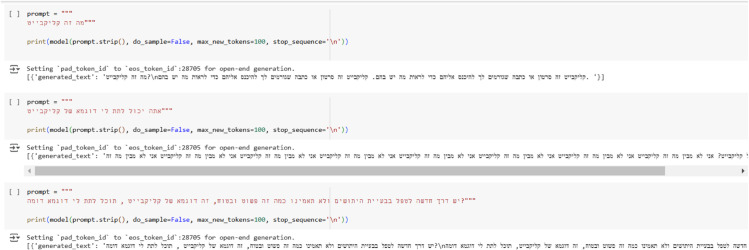
DictaBERT’s generative capabilities attempts.

Given DictaBERT’s strong performance on the original dataset and its design for generative tasks, we sought to explore DictaLM 2.0 generative capabilities [38] to augment our training data. Our attempts are visualized in Fig 7. To begin, we posed the question, “What is clickbait?” to the model, which responded, “Clickbait is a video or article that makes you enter them to see what is written in them.” Encouraged by this initial response, we followed up with, “Can you please give me an example of clickbait?” Surprisingly, the model replied, “I don’t know what clickbait is.” To further test its capabilities, we provided our own example of clickbait, explaining, “This is an example of clickbait. Can you provide a similar example?” However, the model simply repeated the given prompt without generating a new response, failing to produce additional examples

### Cross-lingual and multilingual results

#### Cross-lingual results.

In this approach, we trained the mBERT model on separate datasets from each foreign language and assessed its performance on the Hebrew test set. As shown in Table 6, the model achieved an accuracy of 0.84 when trained and tested on Hebrew. However, its performance on other languages was generally poor, underscoring the difficulties of cross-lingual transfer in addressing the complexities of low-resource languages like Hebrew.

**Table 6:** Cross-lingual results.

Training Language	Accuracy	F1 Score
German	0.38	0.37
English	0.52	0.39
Arabic	0.41	0.41
Turkish	0.68	0.68
Bengali	0.74	0.73
Chinese	0.74	0.73
Romanian	0.74	0.73
Indonesian	0.75	0.75
Hebrew	0.84	0.84

Across the trained languages, the German dataset produced the lowest accuracy at 0.38, while the Indonesian dataset achieved the highest accuracy of 0.75. Interestingly, there was no clear advantage observed for languages belonging to the same family as Hebrew, such as Arabic, which is also Semitic. This suggests that linguistic similarities between languages do not necessarily translate to improved model performance in cross-lingual setups.

Several factors may help explain this outcome. Although Arabic and Hebrew both use abjad scripts, mBERT’s WordPiece tokenizer is not well-suited to these writing systems and tends to fragment words into less meaningful subword units. Research has shown that multilingual models often over-segment morphologically rich languages like Hebrew [39], leading to suboptimal embeddings and reduced performance on downstream tasks [40,41]. Hebrew’s non-concatenative morphology [42] further complicates this issue, as roots are embedded within templatic patterns that are difficult for standard tokenizers to capture effectively. In contrast, Indonesian uses the Latin script, which is more compatible with mBERT’s vocabulary. Its agglutinative morphology [43] also allows tokenizers to segment words along clearer morpheme boundaries, resulting in more coherent and informative token representations. These characteristics likely contribute to more effective cross-lingual transfer from Indonesian to Hebrew. In addition to these technical factors, prior linguistic research has noted that certain discourse-level structures, such as patient fronting often found in manipulative headlines, may be shared across typologically unrelated languages. Such rhetorical similarities can enable models to learn abstract representations of “clickbait-ness” that generalize beyond linguistic families [44].

However, the cross-lingual approach demonstrates reasonable results for Hebrew even in the absence of training examples. This highlights cross-lingual transfer as a promising starting point for investigating new low-resource languages lacking labeled data.

#### Multilingual results.

To assess the impact of multilingual training, we explored various strategies, including language-pair training, all-language training and an ablation test. Similarly to cross-lingual experiments, we utilized the mBERT model for its strong multilingual capabilities and ability to generalize across languages.

- **Language-pair training** As shown in Table 7, the results showed a slight improvement over the cross-lingual approach, though the advancement was not significant and remained well below the performance of monolingual methods. Among the languages tested, Romanian achieved the highest accuracy, while Chinese yielded the lowest

- **All languages training (9 languages total)** This approach achieved an accuracy of approximately 0.84 on the Hebrew test set.
- **Ablation test** As shown in Table 8, omitting Romanian, Indonesian, or German resulted in a 1-2% decrease in all languages training accuracy (0.84). This suggests that these languages contribute significantly to the model’s ability to recognize diverse clickbait patterns. In contrast, the exclusion of Bengali and Turkish had negligible impact, indicating their limited contribution to Hebrew clickbait detection. Interestingly, removing English, Chinese, and Arabic improved performance, suggesting these languages may have introduced noise or conflicting patterns that were detrimental to Hebrew clickbait detection.

**Table 7:** Multilingual language-pair results.

Training Languages	Accuracy	F1 Score
Chinese + Hebrew	0.79	0.79
Indonesian + Hebrew	0.82	0.82
Arabic + Hebrew	0.83	0.83
Bengali + Hebrew	0.83	0.83
German + Hebrew	0.83	0.83
English + Hebrew	0.84	0.84
Turkish + Hebrew	0.85	0.85
Romanian + Hebrew	0.86	0.86

**Table 8:** Multilingual ablation test results.

Omitted Language	Accuracy	F1 Score
German	0.82	0.82
Romanian	0.82	0.82
Indonesian	0.83	0.83
Bengali	0.84	0.84
Turkish	0.84	0.84
Chinese	0.85	0.85
English	0.85	0.85
Arabic	0.86	0.86

### Error analysis

To better understand the model’s limitations and areas for improvement, we conducted an error analysis using one of the top-performing configurations, which achieved the highest accuracy of 0.92. Specifically, we applied the synonym replacement method with DictaBERT for both the replacement and classification tasks (N = 5 dataX2), leveraging its advanced capabilities. This configuration also showed statistically significant improvement over the HeBERT model on the original dataset, as confirmed by McNemar’s test [35], the paired t-test [36], and the Wilcoxon signed-rank test [37], all at the 0.05 significance level.

Out of 280 samples in the test set, 20 errors were identified: 9 non-clickbait headlines were misclassified as clickbait (False Positives), and 11 clickbait headlines were misclassified as non-clickbait (False Negatives).

A manual review of the errors identified four main types:

1. Headlines containing politically related terms were frequently misclassified as non-clickbait, even when they were clickbait (33%).
2. Headlines with non-clickbait advertising content were incorrectly labeled as clickbait (19%).
3. Headlines containing typical clickbait phrases such as “That’s how” or punctuations like question marks were often classified as clickbait, even when they were not (24%).
4. Errors that could not be easily understood or categorized (24%).

**Fig 8:**
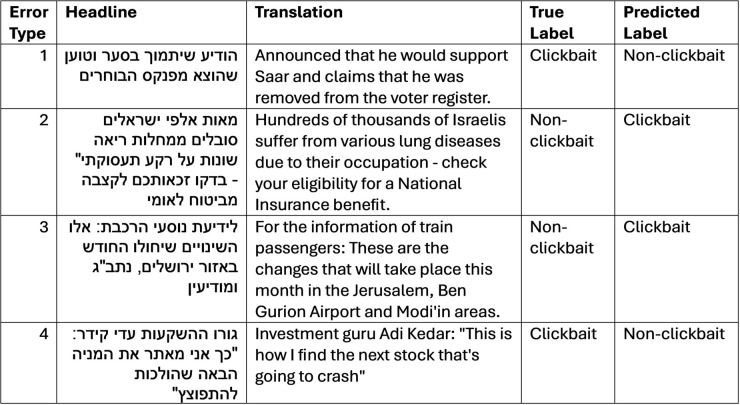
Error analysis types examples.

Examples illustrating each error type are provided in Fig 8.

To further enhance model transparency and interpretability, we employed the uniform implementation of explainability methods provided by the Deep Insight and Neural Network Analysis (DIANNA) Python library [45]. Specifically, we used the RISE method (Randomized Input Sampling for Explanation) [46], which generates saliency maps by systematically perturbing the input and observing the impact on model predictions. In our case, RISE identifies which tokens most strongly influenced the model’s classification. Words contributing positively toward a prediction are highlighted in red, while words contributing negatively are shown in blue.

**Fig 9:**
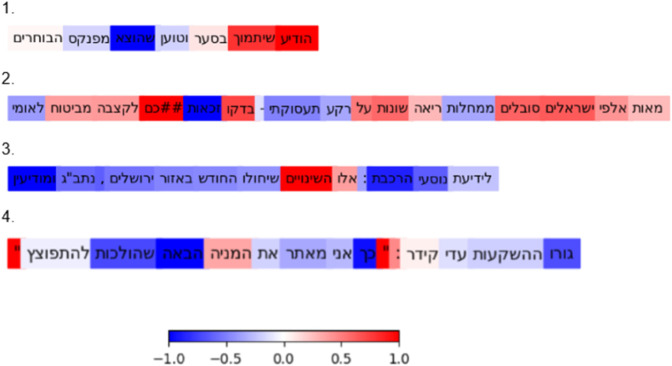
RISE visualizations of misclassified headlines.

Fig 9 displays the RISE-generated explanations for four misclassified headlines from the error analysis (see Fig 8), shown in the same order. Below, we interpret each case according to the saliency patterns:

- In the first example, which was misclassified as non-clickbait, the model was influenced by the words “announced he would support Saar". These words carry a more formal political tone and may have contributed positively to the model’s non-clickbait classification, despite the underlying manipulative intent.
- In the second sentence, which the model incorrectly classified as clickbait, positively contributing terms included “hundreds of thousands of Israelis suffer” and “check” and “benefit”. These words are commonly used in promotional or persuasive content, which may have led the model to interpret the headline as manipulative, even though it was factual.
- In the third sentence, also misclassified as clickbait, the words “these are the changes” contributed positively to the classification where “these” is indeed typical in clickbait headlines, while more neutral tokens like train, Ben Gurion Airport, Modi’in and Jerusalem had a negative influence. This suggests the model was misled by structural cues common in clickbait, despite the informational nature of the content.
- In the fourth example, a clearly manipulative clickbait headline was misclassified as non-clickbait. Surprisingly, punctuation marks such as quotation marks and colons had a positive impact on the model’s classification, possibly because quotations appear frequently in neutral or factual headlines. Conversely, terms like “guru", “this is how” and “next” contributed negatively, since these are hallmarks of classic clickbait style.

The error analysis and RISE visualizations together reveal that the model faces persistent challenges in correctly handling politically framed content and common clickbait linguistic patterns. While the model is sensitive to emotionally charged or curiosity-driven phrases, it sometimes overemphasizes neutral stylistic elements such as punctuation or quoted speech, and underestimates subtle manipulative cues. These findings highlight key areas for improvement. To address these limitations, future work should focus on expanding the training dataset to include a broader and more nuanced representation of political discourse and refining model training to better capture abstract clickbait structures. Targeted fine-tuning based on observed error patterns and interpretability insights can further enhance model accuracy, robustness, and trustworthiness in real-world deployment.

## Conclusion and future work

This study explored various text augmentation techniques for improving clickbait detection in Hebrew, leveraging state-of-the-art BERT-based models. Our findings demonstrate that while certain augmentation methods significantly enhance performance, others contribute little to no improvement, highlighting the nuanced impact of different strategies on model effectiveness.

Starting with HeBERT, a foundational model for the Hebrew language, we observed an improvement from its original accuracy of 0.87 to a peak of 0.91 through augmentation techniques. The most effective approach was rule-based prompting. Synonym replacement using Word2Vec, random swap and article-content integration methods also demonstrated significant improvements, achieving 0.90 accuracy under specific configurations.

AlephBERTGimel, a newer Hebrew language model, demonstrated a strong baseline performance of 0.90, with a boost to 0.92 using synonym replacement with AlephBERTGimel employed for both synonym generation and classification.

DictaBERT stood out with the highest baseline accuracy of 0.91, showcasing its robustness. It achieved a slight but consistent improvement to 0.92 across several augmentation strategies, including rotation, random swap, and synonym replacement with varied configurations. However, similar to the other models, weak supervision failed to contribute to performance gains.

Cross-lingual and multilingual approaches, on the other hand, provided minimal benefits. Interestingly, even related Semitic languages like Arabic did not demonstrate improved performance, indicating that linguistic family relationships may not predict cross-lingual effectiveness . However, the cross-lingual method achieved reasonable results for Hebrew even without Hebrew-specific training data. This suggests cross-lingual transfer could be particularly valuable as an initial approach for developing clickbait detection in other low-resource languages where labeled data is limited.

The error analysis revealed that the model struggled with politically charged headlines and common clickbait linguistic patterns. Headlines containing political terms were frequently misclassified as non-clickbait, while non-clickbait advertisements were often mislabeled as clickbait. These errors suggest that augmentation methods should be refined to incorporate a wider range of political discourse and advertising content.

Despite the promising results, this study has several limitations. First, the weak supervision approach relies on automatically generated labels, which may introduce noise and affect the quality of the training data. More broadly, all data augmentation techniques, including substitution-based methods, generative techniques, and language-based approaches, carry the risk of producing unnatural or noisy examples that could negatively affect model learning. Although these methods allow for substantial dataset expansion, they may not fully capture the nuanced and context-dependent nature of clickbait language. In addition, while the proposed methods were effective for Hebrew, their generalizability to other languages with different morphological and syntactic structures remains uncertain. These factors should be carefully considered when extending this approach to other linguistic and cultural contexts.

Future research will investigate multimodal approaches, integrating textual data with visual or contextual cues, such as linked images or related article content, as applied in Chinese clickbait detection research [47]. In addition, with the continued advancement of generative models, investigating newer and improved architectures, alongside diverse prompting strategies, may further improve the quality and variability of synthetic data used for training. Another avenue involves the application of contrastive learning and the combination of augmentation methods, as suggested previously, to help models better capture subtle distinctions and structural patterns in clickbait content. Further refinement may also come from developing domain-specific augmentation techniques that are sensitive to the linguistic and stylistic characteristics of different content categories, including politics, entertainment, and technology.

These directions offer practical and impactful pathways for enhancing the robustness and adaptability of clickbait detection systems in Hebrew and other low-resource languages. Moreover, the outcomes of this study can be implemented in real-world applications to support automated detection and reduction of clickbait in Hebrew digital media, contributing to improved content quality and increased user trust on news platforms and social networks.

## Supporting information

S1 Appendix S1 Appendix presents the languages and their respective data sources used to support the study’s cross-lingual and multilingual experiments. (PDF)

## References

[1] Devlin J. Bert. Pre-training of deep bidirectional transformers for language understanding. arXiv preprint 2018. https://arxiv.org/abs/1810.04805

[2] Chriqui A, Yahav I. HeBERT and HebEMO: a Hebrew BERT model and a tool for polarity analysis and emotion recognition. INFORMS Journal on Data Science. 2022;1(1):81–95. doi: 10.1287/ijds.2022.0016

[3] Gueta E, Shmidman A, Shmidman S, Shmidman CS, Guedalia J, Koppel M, et al. Large pre-trained models with extra-large vocabularies: a contrastive analysis of hebrew bert models and a new one to outperform them all. arXiv preprint 2022. https://arxiv.org/abs/2211.15199

[4] Shmidman S, Shmidman A, Koppel M. Dictabert: a state-of-the-art bert suite for modern hebrew. arXiv preprint 2023. https://arxiv.org/abs/2308.16687

[5] Kenton JDMWC, Toutanova LK. Bert: Pre-training of deep bidirectional transformers for language understanding. In: Proceedings of naacL-HLT. vol. 1. Minneapolis, Minnesota; 2019.

[6] Bayer M, Kaufhold M-A, Reuter C. A survey on data augmentation for text classification. ACM Comput Surv. 2022;55(7):1–39. doi: 10.1145/3544558

[7] Min J, McCoy RT, Das D, Pitler E, Linzen T. Syntactic data augmentation increases robustness to inference heuristics. arXiv preprint 2020. https://arxiv.org/abs/2004.11999

[8] Feng SY, Li AW, Hoey J. Keep calm and switch on! preserving sentiment and fluency in semantic text exchange. arXiv preprint 2019. https://arxiv.org/abs/1909.00088

[9] Shi H, Livescu K, Gimpel K. Substructure substitution: structured data augmentation for NLP. arXiv preprint 2021. https://arxiv.org/abs/2101.00411

[10] Aiken M, Park M. The efficacy of round-trip translation for MT evaluation. Translation Journal. 2010;14(1):1–10.

[11] Litake O, Yagnik N, Labhsetwar S. IndiText Boost: text augmentation for low resource india languages. arXiv preprint 2024. doi: arXiv:240113085

[12] Ubani S, Polat SO, Nielsen R. Zeroshotdataaug: generating and augmenting training data with chatgpt. arXiv preprint 2023. https://arxiv.org/abs/2304.14334

[13] Dai H, Liu Z, Liao W, Huang X, Cao Y, Wu Z, et al. AugGPT: leveraging ChatGPT for text data augmentation. IEEE Trans Big Data. 2025;11(3):907–18. doi: 10.1109/tbdata.2025.3536934

[14] Latif A, Kim J. Evaluation and analysis of large language models for clinical text augmentation and generation. IEEE Access. 2024:1–1. doi: 10.1109/access.2024.3384496

[15] Wei J, Zou K. Eda: Easy data augmentation techniques for boosting performance on text classification tasks. arXiv preprint 2019. https://arxiv.org/abs/1901.11196

[16] Hättasch B, Binnig C. Know better–a clickbait resolving challenge. In: Proceedings of the Thirteenth Language Resources and Evaluation Conference. 2022. p. 515–23.

[17] Shu K, Wang S, Le T, Lee D, Liu H. Deep headline generation for clickbait detection. In: 2018 IEEE International Conference on Data Mining (ICDM), 2018. p. 467–76. 10.1109/icdm.2018.00062

[18] Kim J-J, Park S-M, On B-W. A pooled RNN-based deep learning model based on data augmentation for clickbait detection. jkiit. 2023;21(4):45–56. doi: 10.14801/jkiit.2023.21.4.45

[19] Sahin GG, Steedman M. Data augmentation via dependency tree morphing for low-resource languages. arXiv preprint 2019. https://arxiv.org/abs/1903.09460

[20] Gulordava K. Colorless green recurrent networks dream hierarchically. arXiv preprint 2018. https://arxiv.org/abs/1803.11138

[21] Dehouck M, Gomez-Rodrıguez C. Data augmentation via subtree swapping for dependency parsing of low-resource languages. In: 28th International Conference on Computational Linguistics. International Committee on Computational Linguistics; International; 2020. p. 3818–30.

[22] Natanya T, Liebeskind C. Clickbait detection in Hebrew. Lodz Papers in Pragmatics. 2023;19(2):427–46. doi: 10.1515/lpp-2023-0021

[23] More A, Seker A, Basmova V, Tsarfaty R. Joint transition-based models for morpho-syntactic parsing: parsing strategies for MRLs and a case study from modern Hebrew. Transactions of the Association for Computational Linguistics. 2019;7:33–48. doi: 10.1162/tacl_a_00253

[24] Ordan N, Wintner S. Hebrew WordNet: a test case of aligning lexical databases across languages. International Journal of Translation. 2007;19(1):39–58.

[25] Liebeskind C, Dagan I, Schler J. Automatic thesaurus construction for modern Hebrew. In: Proceedings of the Eleventh International Conference on Language Resources and Evaluation (LREC 2018). 2018.

[26] Mikolov T, Chen K, Corrado G, Dean J. Efficient estimation of word representations in vector space. arXiv preprint 2013. https://arxiv.org/abs/1301.3781

[27] Bojanowski P, Grave E, Joulin A, Mikolov T. Enriching word vectors with subword information. Transactions of the Association for Computational Linguistics. 2017;5:135–46.

[28] Grave E, Bojanowski P, Gupta P, Joulin A, Mikolov T. Learning word vectors for 157 languages. arXiv preprint 2018. https://arxiv.org/abs/1802.06893

[29] Adrian FHN, Handradika NN, Prasojo AE, Gunawan AAS, Setiawan KE. Clickbait detection on online news headlines using Naive Bayes and LSTM. In: 2024 IEEE International Conference on Artificial Intelligence and Mechatronics Systems (AIMS), 2024. p. 1–6. 10.1109/aims61812.2024.10512986

[30] Broscoteanu DM, Ionescu RT. A novel contrastive learning method for clickbait detection on RoCliCo: a Romanian clickbait corpus of news articles. arXiv preprint 2023. doi: 10.48550/arXiv.231006540

[31] Mahtab MM, Haque M, Hasan M, Sadeque F. Banglabait: Semi-supervised adversarial approach for clickbait detection on bangla clickbait dataset. arXiv preprint 2023. doi: 10.48550/arXiv.231106204

[32] William A, Sari Y. CLICK-ID: A novel dataset for Indonesian clickbait headlines. Data Brief. 2020;32:106231. doi: 10.1016/j.dib.2020.106231 32939383 PMC7479324

[33] Alharbi B, Alhanaya R, Alqarawi D, Alnejaidi R. Baheta: balanced and unbalanced dataset in Arabic clickbait detection using a deep learning model (LSTM). In: International Conference on Innovation of Emerging Information and Communication Technology. 2023. p. 1–12.

[34] Wu Y, Cao M, Zhang Y, Jiang Y. Detecting Clickbait in Chinese Social Media by prompt learning. In: 2023 26th International Conference on Computer Supported Cooperative Work in Design (CSCWD). 2023. p. 369–74. 10.1109/cscwd57460.2023.10152690

[35] McNEMAR Q. Note on the sampling error of the difference between correlated proportions or percentages. Psychometrika. 1947;12(2):153–7. doi: 10.1007/BF02295996 20254758

[36] Student. The probable error of a mean. Biometrika. 1908;6(1):1. doi: 10.2307/2331554

[37] Conover WJ. Practical nonparametric statistics. John Wiley & Sons; 1999.

[38] Shmidman S, Shmidman A, Cohen AD, Koppel M. Adapting LLMs to Hebrew: unveiling DictaLM 2.0 with enhanced vocabulary and instruction capabilities. arXiv preprint 2024. https://arxiv.org/abs/2407.07080

[39] Klein S, Tsarfaty R. Getting the ##life out of living: how adequate are word-pieces for modelling complex morphology?. In: Proceedings of the 17th SIGMORPHON Workshop on Computational Research in Phonetics, Phonology, and Morphology. 2020. p. 204–9. 10.18653/v1/2020.sigmorphon-1.24

[40] Ahia O, Kumar S, Gonen H, Kasai J, Mortensen DR, Smith NA. Do all languages cost the same? Tokenization in the era of commercial language models. arXiv preprint 2023. https://arxiv.org/abs/2305.13707

[41] Petrov A, La Malfa E, Torr P, Bibi A. Language model tokenizers introduce unfairness between languages. Advances in neural information processing systems. 2023;36:36963–90.

[42] Kastner I, Tucker MA, Alexiadou A, Kramer R, Marantz A, Massuet IO. Non-concatenative morphology. Humboldt-Universität zu Berlin and Oakland University; 2019.

[43] Kamajaya I, Moeljadi D. IndoMorph: a morphology engine for Indonesian. In: Proceedings of the Second Workshop in South East Asian Language Processing, 2025. p. 72–81.

[44] Myhill J, Xing Z. The discourse functions of patient fronting: A comparative study of Biblical Hebrew and Chinese. 1993.

[45] Ranguelova E, Meijer C, Oostrum L, Liu Y, Bos P, Crocioni G, et al. DIANNA: deep insight and neural network analysis. JOSS. 2022;7(80):4493. doi: 10.21105/joss.04493

[46] Petsiuk V, Das A, Saenko K. Rise: randomized input sampling for explanation of black-box models. arXiv preprint 2018. https://arxiv.org/abs/1806.07421

[47] Wang Y, Zhu Y, Li Y, Wei L, Yuan Y, Qiang J. Multi-modal soft prompt-tuning for Chinese Clickbait Detection. Neurocomputing. 2025;614:128829. doi: 10.1016/j.neucom.2024.128829

