# Supplementary material for: Towards better Hebrew clickbait detection: Insights from BERT and data augmentation
Source: PLoS One. 2025 Nov 6;20(11):e0332342. doi: 10.1371/journal.pone.0332342 (PMC12591409; doi:10.1371/journal.pone.0332342)
Supplement: S1 Appendix — (PDF) [file pone.0332342.s001.pdf]

S1 Appendix

The datasets in S1 Table 1 were utilized to explore cross-lingual and multilingual training methods in the study.

S1 Table 1. Language-based methods datasets

Language	Source
Turkish	https://www.kaggle.com/code/datafan07/clickbait-news-classification-using-sadedegel
German	https://github.com/youurt/german_clickbaits_tensorflow_js/blob/main/01_Datenaquise/ROHDATEN.csv
English	http://kaggle.com/datasets/amananandrai/clickbait-dataset?resource=download
Romanian	https://github.com/ralucaginga/ClickbaitSciTechRO/blob/main/dataset.xlsx
Bengali	https://www.kaggle.com/datasets/motaharmahtab/banglabait-bangla-clickbait-dataset?resource=download
Indonesian	https://github.com/ruzcmc/ClickbaitIndo-textclassifier
Chinese	https://github.com/WeSeewy/Chinese-Clickbait/blob/main/data/news.txt
Arabic	https://github.com/RazanALhanaya/Clickbait-Fake-News-Dataset/blob/main/clickbaitNew%2BFakeNews%20(1).xlsx
Hebrew	https://github.com/Talya-Natania/Clickbait-Data
